# Supplementary material for: Hypertensive disorders of pregnancy and the risk of chronic kidney disease: A Swedish registry-based cohort study
Source: PLoS Med. 2020 Aug 14;17(8):e1003255. doi: 10.1371/journal.pmed.1003255 (PMC7428061; doi:10.1371/journal.pmed.1003255)
Supplement: S1 Fig — (DOCX) [file pmed.1003255.s001.docx]

**N=4,073,947**

Total pregnancies recorded in Sweden from 1 January 1973 to 31 December 2012

**N=347,393**

Pregnancies excluded from dataset for the following reasons:

| Pre-pregnancy diseases | |  |
| --- | --- | --- |
|  | *Renal disease (any)* | 16,396 |
|  | *Diabetes* | 18,564 |
|  | *Chronic hypertension* | 10,758 |
|  | *Cardiovascular disease* | 3,053 |
|  | *Systemic lupus erythematosus* | 2,462 |
|  | *Systemic sclerosis* | 41 |
|  | *Coagulopathy* | 4,231 |
|  | *Haemoglobinopathy* | 1,514 |
|  | *Vasculitis* | 63 |
| Post-pregnancy disease | |  |
|  | *Renal disease within 3 months of last pregnancy* | 15,200 |
| Stillbirths | | 14,107 |
| Multiple pregnancy | | 148,339 |
| Implausible or incomplete information on date of delivery | | 312 |
| Implausible birth weight for gestational age | | 13,223 |
| Died or emigrated before date of first delivery recorded | | 99,130 |

**N=3,726,554**

Eligible pregnancies in Sweden from 1 January 1973 to 31 December 2012

**S1 Figure. Flow chart illustrating construction of study cohort**
