# Supplementary material for: Hypertensive disorders of pregnancy and the risk of chronic kidney disease: A Swedish registry-based cohort study
Source: PLoS Med. 2020 Aug 14;17(8):e1003255. doi: 10.1371/journal.pmed.1003255 (PMC7428061; doi:10.1371/journal.pmed.1003255)
Supplement: S1 Table — (DOCX) [file pmed.1003255.s002.docx]

**S1 Table. ICD codes used for disease definitions**

|  | **ICD-8 codes**  **(1973-1986)** | **ICD-9 codes**  **(1987-1996)** | **ICD-10 codes**  **(1997-2013)** |
| --- | --- | --- | --- |
| Any pre-existing chronic/end-stage kidney disease, congenital or genetic causes of renal disease (for exclusion) | 403-404, 580-589, 753 | 403-404, 580-589, 753, V42A, V45B, V56A, V56W | N00-N08, N10-N19, P960, Q271, Q272, Q60-Q63, Q878, Z49, Z992, Z940, T861 |
| Chronic kidney disease (CKD)  (Overall outcome) | 250D, 403-404, 581-583, 58499, 585-588 | 250D, 403-404, 581-583, 585-588, V42A, V45B, V56A, V56W | E102, E112, I12-I13, I150-I151, N01-N06, N08, N11-N13, N15-16, N18-19, Z49, Z992, Z940, T861 |
| Tubulointerstitital CKD | - | - | N11-N12, N15-N16 |
| Glomerular/proteinuric CKD | 581-583 | 581-583 | N01-N06, N08 |
| Hypertensive CKD | 403-404 | 403-404 | I12-I13, I150-I151 |
| Diabetic CKD | 250D | 250D | E102, E112 |
| Other/unspecified CKD | 58499, 585-588 | 585-588, V42A, V45B, V56A, V56W | N13, N18-N19, Z49, Z992, Z940, T861 |
| Cardiovascular disease | 393-398  410-436 | 393-398  410-436 | I16-I64  G45 |
| Essential hypertension | 401-405 | 401-405 | I10-I15 |
| Diabetes (type 1 or type 2) | 250 | 250 | E10-E14 |
| Systemic lupus erythematosus | 73410 | 710A | M32 |
| Systemic sclerosis | 73400-73409 | 710B | M34 |
| Vasculitis | 446 | 446 | M31 |
| Hemoglobinopathies | 282-283 | 282-283 | D56-D59 |
| Coagulopathies | None | None | D68 |
| Preeclampsia | 63700, 63703, 63704, 63709, 63710, 63719, 63790, 63799 | 642E, 642F, 642G | O140, O141, O141A,  O141B, O141C, O141X, O142, O149, O150, O151, O152, O159 |
| Gestational hypertension | 63701 | 642D, 642X | O130-O139 |
| Gestational diabetes | - | 648W | O244 |
