# Supplementary material for: Hypertensive disorders of pregnancy and the risk of chronic kidney disease: A Swedish registry-based cohort study
Source: PLoS Med. 2020 Aug 14;17(8):e1003255. doi: 10.1371/journal.pmed.1003255 (PMC7428061; doi:10.1371/journal.pmed.1003255)
Supplement: S2 Fig — (DOCX) [file pmed.1003255.s003.docx]

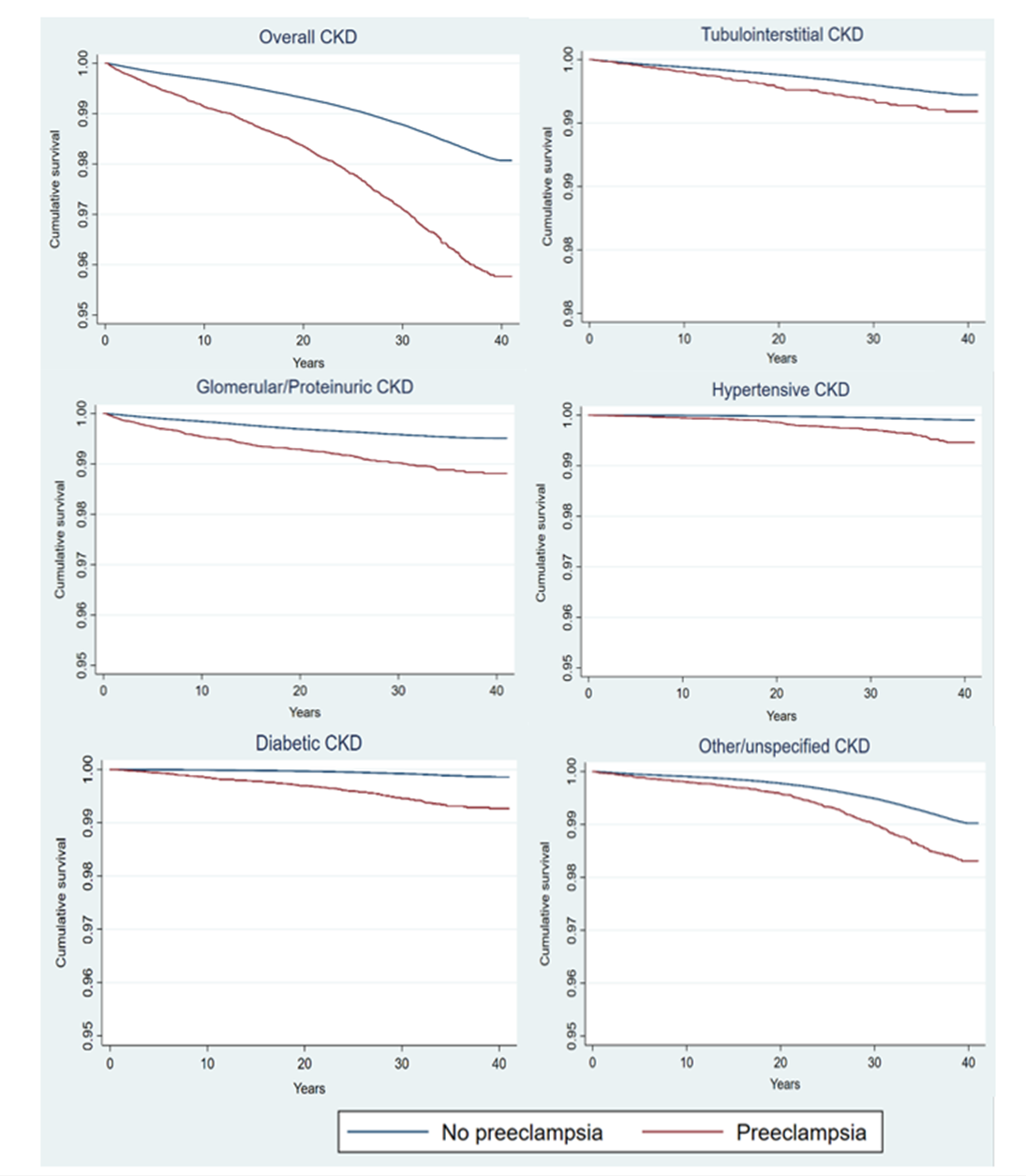


| **Overall CKD** | **0-10 years** | **10-20 years** | **20-30 years** | **30-41 years** |
| --- | --- | --- | --- | --- |
| ***No preeclampsia*** |  |  |  |  |
| Number at risk | 1833492 | 1391945 | 1017878 | 591091 |
| Number of events | 5309 | 4447 | 4269 | 2908 |
| ***Preeclampsia*** |  |  |  |  |
| Number at risk | 90917 | 71306 | 50031 | 27568 |
| Number of events | 601 | 419 | 347 | 177 |

**S2 Figure. Kaplan-Meier survival curves for** **risk of chronic kidney disease (overall and subtypes) among women whose first live birth occurred between 1973 and 2012 in Sweden, by exposure to preeclampsia.**
