# Supplementary material for: Hypertensive disorders of pregnancy and the risk of chronic kidney disease: A Swedish registry-based cohort study
Source: PLoS Med. 2020 Aug 14;17(8):e1003255. doi: 10.1371/journal.pmed.1003255 (PMC7428061; doi:10.1371/journal.pmed.1003255)
Supplement: S2 Table — (DOCX) [file pmed.1003255.s004.docx]

**S2 Table. STROBE Statement—checklist of items that should be included in reports of observational studies**

| Item No | | | Recommendation | Location |
| --- | --- | --- | --- | --- |
| **Title and abstract** | | 1 | (*a*) Indicate the study’s design with a commonly used term in the title or the abstract | Title & Abstract |
|  |  |  | (*b*) Provide in the abstract an informative and balanced summary of what was done and what was found | Abstract (Methods and findings) |
| Introduction | | | |  |
| Background/rationale | | 2 | Explain the scientific background and rationale for the investigation being reported | Background (Paragraphs 1-2) |
| Objectives | | 3 | State specific objectives, including any prespecified hypotheses | Background (Paragraph 3) |
| Methods | | | |  |
| Study design | | 4 | Present key elements of study design early in the paper | Methods (Study population section) |
| Setting | | 5 | Describe the setting, locations, and relevant dates, including periods of recruitment, exposure, follow-up, and data collection | Methods (Study population section) |
| Participants | | 6 | (*a*) *Cohort study*—Give the eligibility criteria, and the sources and methods of selection of participants. Describe methods of follow-up | Methods (Study population section & Statistical analysis section, first paragraph) |
|  |  |  | (*b*) *Cohort study*—For matched studies, give matching criteria and number of exposed and unexposed | NA |
| Variables | | 7 | Clearly define all outcomes, exposures, predictors, potential confounders, and effect modifiers. Give diagnostic criteria, if applicable | Outcomes: Methods (Outcome variables - Chronic kidney disease section)  Exposures: Methods (Exposure variables section)  Potential confounders: Methods (Outcome variables - Covariates section & Statistical analysis section, second paragraph)  Effect modifiers: Methods (Statistical analysis section, third paragraph) |
| Data sources/ measurement | | 8* | For each variable of interest, give sources of data and details of methods of assessment (measurement). Describe comparability of assessment methods if there is more than one group | Outcomes: Methods (Outcome variables - Chronic kidney disease section & Covariates section)  Exposures: Methods (Exposure variables section) |
| Bias | | 9 | Describe any efforts to address potential sources of bias | Methods (Study population, first paragraph & Exposure variables, second paragraph & Outcome variables, Chronic kidney disease section, first paragraph) |
| Study size | | 10 | Explain how the study size was arrived at | Methods (Study population section) & Supplementary Figure S1 |
| Quantitative variables | | 11 | Explain how quantitative variables were handled in the analyses. If applicable, describe which groupings were chosen and why | Methods (Exposure variables section & Outcome variables section) |
| Statistical methods | | 12 | (*a*) Describe all statistical methods, including those used to control for confounding | Methods (Statistical analysis section) |
|  |  |  | (*b*) Describe any methods used to examine subgroups and interactions | Methods (Statistical analysis section, second and third paragraphs) |
|  |  |  | (*c*) Explain how missing data were addressed | Methods (Outcome variables - Covariates section, first paragraph & Discussion, Strengths & Limitations section, fourth paragraph) |
|  |  |  | (*d*) *Cohort study*—If applicable, explain how loss to follow-up was addressed  *Case-control study*—If applicable, explain how matching of cases and controls was addressed  *Cross-sectional study*—If applicable, describe analytical methods taking account of sampling strategy | Methods (Study population section & Statistical analysis section, first paragraph) |
|  |  |  | (*e*) Describe any sensitivity analyses | Methods (Statistical analysis section, third paragraph) |
| Results | | | |  |
| Participants | 13* | (a) Report numbers of individuals at each stage of study—eg numbers potentially eligible, examined for eligibility, confirmed eligible, included in the study, completing follow-up, and analysed | | Methods (Statistical analysis section) & Supplementary Figure S1 |
|  |  | (b) Give reasons for non-participation at each stage | | Methods (Study population section & Statistical analysis section, first and third paragraphs) & Supplementary Figure S1 |
|  |  | (c) Consider use of a flow diagram | | Supplementary Figure S1 |
| Descriptive data | 14* | (a) Give characteristics of study participants (eg demographic, clinical, social) and information on exposures and potential confounders | | Results (Paragraphs 1-3) & Table 1 |
|  |  | (b) Indicate number of participants with missing data for each variable of interest | | Results, Table 1 |
|  |  | (c) *Cohort study*—Summarise follow-up time (eg, average and total amount) | | Results (Paragraphs 1 & 3) |
| Outcome data | 15* | *Cohort study*—Report numbers of outcome events or summary measures over time | | Results (Paragraph 1 & Tables 2-5) |
|  |  | *Case-control study—*Report numbers in each exposure category, or summary measures of exposure | | NA |
|  |  | *Cross-sectional study—*Report numbers of outcome events or summary measures | | NA |
| Main results | 16 | (*a*) Give unadjusted estimates and, if applicable, confounder-adjusted estimates and their precision (eg, 95% confidence interval). Make clear which confounders were adjusted for and why they were included | | Methods (Outcome variables section & Statistical analysis section, first and second paragraph)  Results (Preeclampsia section, Sensitivity analysis section, Gestational hypertension section, Tables 2-5) |
|  |  | (*b*) Report category boundaries when continuous variables were categorized | | Results (Table 1) |
|  |  | (*c*) If relevant, consider translating estimates of relative risk into absolute risk for a meaningful time period | | Supplementary Figure S2 |
| Other analyses | 17 | Report other analyses done—eg analyses of subgroups and interactions, and sensitivity analyses **Page 12-13, Page 17-18 (Table 5), Online Supplement** | | Results (Sensitivity analysis section, Gestational hypertension section) & Supplementary Tables S3-S10 |
| Discussion | | | |  |
| Key results | 18 | Summarise key results with reference to study objectives | | Discussion (Paragraphs 1-3) |
| Limitations | 19 | Discuss limitations of the study, taking into account sources of potential bias or imprecision. Discuss both direction and magnitude of any potential bias | | Discussion (Strengths & Limitations section) |
| Interpretation | 20 | Give a cautious overall interpretation of results considering objectives, limitations, multiplicity of analyses, results from similar studies, and other relevant evidence | | Discussion (Paragraphs 2-6) |
| Generalisability | 21 | Discuss the generalisability (external validity) of the study results | | Discussion (Strengths & Limitations section, third and fifth paragraphs) |
| Other information | | | |  |
| Funding | 22 | Give the source of funding and the role of the funders for the present study and, if applicable, for the original study on which the present article is based | | Financial disclosure section |

*Give information separately for cases and controls in case-control studies and, if applicable, for exposed and unexposed groups in cohort and cross-sectional studies.
