# Supplementary material for: Hypertensive disorders of pregnancy and the risk of chronic kidney disease: A Swedish registry-based cohort study
Source: PLoS Med. 2020 Aug 14;17(8):e1003255. doi: 10.1371/journal.pmed.1003255 (PMC7428061; doi:10.1371/journal.pmed.1003255)
Supplement: S3 Table — (DOCX) [file pmed.1003255.s005.docx]

**S3 Table.** **Time to diagnosis of chronic kidney disease (CKD) subtypes among women whose first live birth occurred between 1973 and 2012 in Sweden, stratified by exposure to preeclampsia (n=1,924,409)**

|  | | **n** | **Time to diagnosis (years)** | |
| --- | --- | --- | --- | --- |
|  | |  | **Median (IQR)** | **Log-rank, p** |
| **Overall CKD** | |  |  |  |
| No preeclampsia | | 16,933 | 16.9 (7.3-26.6) | <0.001 |
| Exposed to preeclampsia | | 1,544 | 14.2 (4.7-24.7) |  |
| **1.** | **Tubulointerstitial CKD** |  |  |  |
|  | No preeclampsia | 2,629 | 14.7 (5.9-24.2) | <0.001 |
|  | Exposed to preeclampsia | 184 | 13.4 (5.5-20.5) |  |
| **2.** | **Glomerular/proteinuric CKD** |  |  |  |
|  | No preeclampsia | 5,568 | 11.2 (4.6-18.5) | <0.001 |
|  | Exposed to preeclampsia | 500 | 7.7 (2.0-15.7) |  |
| **3.** | **Hypertensive CKD** |  |  |  |
|  | No preeclampsia | 667 | 22.7 (15.3-30.2) | <0.001 |
|  | Exposed to preeclampsia | 130 | 20.6 (11.0-29.2) |  |
| **4.** | **Diabetic CKD** |  |  |  |
|  | No preeclampsia | 1,011 | 23.0 (13.9-30.3) | <0.001 |
|  | Exposed to preeclampsia | 215 | 15.6 (6.6-25.2) |  |
| **5.** | **Other/unspecified CKD** |  |  |  |
|  | No preeclampsia | 7,058 | 22.2 (11.6-30.1) | <0.001 |
|  | Exposed to preeclampsia | 515 | 21.3 (8.4-28.4) |  |
