# Supplementary material for: Hypertensive disorders of pregnancy and the risk of chronic kidney disease: A Swedish registry-based cohort study
Source: PLoS Med. 2020 Aug 14;17(8):e1003255. doi: 10.1371/journal.pmed.1003255 (PMC7428061; doi:10.1371/journal.pmed.1003255)
Supplement: S4 Table — HRs represent results of Cox regression models for associations between preeclampsia ± SGA and maternal CKD. Preeclampsia was a time-dependent variable. Fully adjusted models controlled for maternal age, country of origin, education level, parity, maternal BMI, smoking in pregnancy, exposure to gestational diabetes, and exposure to gestational hypertension. Models were stratified by year of delivery. *All p < 0.001. CKD, chronic kidney disease; HR, hazard ratio; SGA, small for gestational age. (DOCX) [file pmed.1003255.s006.docx]

**S4 Table. Hazard ratios for maternal chronic kidney disease by history of preeclampsia and small for gestational age, among women whose first live birth occurred between 1987 and 2012 in Sweden (n=1,127,798)**

|  | | **n** | **Age-adjusted** | **Fully adjusted** |
| --- | --- | --- | --- | --- |
|  | |  | **HR (95% CI*)** | **HR (95% CI*)** |
| **Overall CKD** | |  |  |  |
| No preeclampsia, no SGA | | 5,184 | 1.0 | 1.0 |
| Preeclampsia only | | 511 | 2.34 (2.14-2.57) | 2.29 (2.09-2.52) |
| SGA only | | 294 | 1.53 (1.36-1.72) | 1.38 (1.22-1.55) |
| Preeclampsia & SGA | | 87 | 2.21 (1.79-2.74) | 2.11 (1.71-2.61) |
| **1.** | **Tubulointerstitial CKD** |  |  |  |
|  | No preeclampsia, no SGA | 1,192 | 1.0 | 1.0 |
|  | Preeclampsia only | 84 | 1.65 (1.32-2.06) | 1.68 (1.35-2.10) |
|  | SGA only | 56 | 1.30 (0.99-1.70) | 1.11 (0.85-1.46) |
|  | Preeclampsia & SGA | 16 | 1.79 (1.09-2.94) | 1.71 (1.05-2.81) |
| **2.** | **Glomerular/proteinuric CKD** |  |  |  |
|  | No preeclampsia, no SGA | 1,498 | 1.0 | 1.0 |
|  | Preeclampsia only | 160 | 2.62 (2.22-3.08) | 2.61 (2.21-3.08) |
|  | SGA only | 97 | 1.74 (1.42-2.14) | 1.58 (1.28-1.94) |
|  | Preeclampsia & SGA | 26 | 2.34 (1.59-3.45) | 2.26 (1.53-3.33) |
| **3.** | **Hypertensive CKD** |  |  |  |
|  | No preeclampsia, no SGA | 108 | 1.0 | 1.0 |
|  | Preeclampsia only | 14 | 3.17 (1.81-5.53) | 2.99 (1.70-5.29) |
|  | SGA only | 7 | 1.57 (0.73-3.37) | 1.38 (0.64-2.97) |
|  | Preeclampsia & SGA | 8 | 9.18 (4.47-18.84) | 8.64 (4.19-17.81) |
| **4.** | **Diabetic CKD** |  |  |  |
|  | No preeclampsia, no SGA | 232 | 1.0 | 1.0 |
|  | Preeclampsia only | 89 | 9.07 (7.10-11.59) | 7.01 (5.44-9.05) |
|  | SGA only | 12 | 1.30 (0.73-2.33) | 1.19 (0.67-2.14) |
|  | Preeclampsia & SGA | 8 | 4.31 (2.13-8.72) | 3.80 (1.87-7.70) |
| **5.** | **Other/unspecified CKD** |  |  |  |
|  | No preeclampsia, no SGA | 2,154 | 1.0 | 1.0 |
|  | Preeclampsia only | 164 | 1.79 (1.53-2.10) | 1.76 (1.50-2.07) |
|  | SGA only | 122 | 1.53 (1.28-1.84) | 1.41 (1.18-1.70) |
|  | Preeclampsia & SGA | 29 | 1.76 (1.22-2.54) | 1.69 (1.17-2.44) |

Hazard ratios represent results of Cox regression models for associations between preeclampsia +/- small for gestational age and maternal chronic kidney disease. Preeclampsia was a time-dependent variable. Fully adjusted models controlled for maternal age, country of origin, education level, parity, maternal BMI, smoking in pregnancy, exposure to gestational diabetes, and exposure to gestational hypertension. Models were stratified by year of delivery. Abbreviations: CI, confidence interval; CKD, chronic kidney disease; HR, hazard ratio; SGA, small for gestational age. *All p<0.001
