# Supplementary material for: Hypertensive disorders of pregnancy and the risk of chronic kidney disease: A Swedish registry-based cohort study
Source: PLoS Med. 2020 Aug 14;17(8):e1003255. doi: 10.1371/journal.pmed.1003255 (PMC7428061; doi:10.1371/journal.pmed.1003255)
Supplement: S6 Table — *All p < 0.001. **Exact number not reported as cell count ≤ 5. (DOCX) [file pmed.1003255.s008.docx]

**S6 Table. Hazard ratios for maternal chronic kidney disease by history of recurrent preeclampsia, among women whose first live birth occurred between 1987 and 2012 in Sweden (n=548,621)**

|  | | **N** | **Age-adjusted** | **Fully adjusted** |
| --- | --- | --- | --- | --- |
|  | |  | **HR (95% CI*)** | **HR (95% CI*)** |
| **Overall CKD** | |  |  |  |
| Two pregnancies without preeclampsia | | 2,583 | 1.0 | 1.0 |
| Two pregnancies, one episode preeclampsia | | 258 | 2.14 (1.88-2.43) | 2.09 (1.83-2.38) |
| Two pregnancies, two episodes preeclampsia | | 41 | 2.76 (2.03-3.76) | 2.66 (1.95-3.63) |
| **1.** | **Tubulointerstitial CKD** |  |  |  |
|  | Two pregnancies without preeclampsia | 580 | 1.0 |  |
|  | Two pregnancies, one episode preeclampsia | 42 | 1.52 (1.12-2.09) | 1.54 (1.12-2.11) |
|  | Two pregnancies, two episodes preeclampsia | ** | 1.49 (0.62-3.58) | 1.47 (0.61-3.55) |
| **2.** | **Glomerular/proteinuric CKD** |  |  |  |
|  | Two pregnancies without preeclampsia | 761 | 1.0 | 1.0 |
|  | Two pregnancies, one episode preeclampsia | 93 | 2.36 (2.12-3.26) | 2.68 (2.15-3.33) |
|  | Two pregnancies, two episodes preeclampsia | 15 | 3.45 (2.07-5.76) | 3.44 (2.06-5.74) |
| **3.** | **Hypertensive CKD** |  |  |  |
|  | Two pregnancies without preeclampsia | 50 | 1.0 | 1.0 |
|  | Two pregnancies, one episode preeclampsia | 11 | 4.87 (2.53-9.36) | 4.19 (2.14-8.20) |
|  | Two pregnancies, two episodes preeclampsia | ** | 7.22 (1.76-29.72) | 6.65 (1.60-27.57) |
| **4.** | **Diabetic CKD** |  |  |  |
|  | Two pregnancies without preeclampsia | 102 | 1.0 | 1.0 |
|  | Two pregnancies, one episode preeclampsia | 35 | 7.36 (5.01-10.81) | 6.08 (4.11-9.02) |
|  | Two pregnancies, two episodes preeclampsia | 7 | 11.64 (5.41-25.05) | 8.94 (4.11-19.43) |
| **5.** | **Other/unspecified CKD** |  |  |  |
|  | Two pregnancies without preeclampsia | 1,090 | 1.0 | 1.0 |
|  | Two pregnancies, one episode preeclampsia | 77 | 1.51 (1.20-1.91) | 1.45 (1.15-1.83) |
|  | Two pregnancies, two episodes preeclampsia | 12 | 1.92 (1.09-3.39) | 1.86 (1.05-3.29) |

*All p<0.001. **Exact number not reported as cell count ≤5
