# Supplementary material for: Hypertensive disorders of pregnancy and the risk of chronic kidney disease: A Swedish registry-based cohort study
Source: PLoS Med. 2020 Aug 14;17(8):e1003255. doi: 10.1371/journal.pmed.1003255 (PMC7428061; doi:10.1371/journal.pmed.1003255)
Supplement: S7 Table — HRs represent separate Cox regression models for associations between preeclampsia and maternal CKD. Preeclampsia was a time-dependent variable. Fully adjusted models controlled for maternal age, country of origin, education level, parity, maternal BMI, smoking in pregnancy, exposure to gestational diabetes, and exposure to gestational hypertension. Models were stratified by year of delivery. *All p < 0.001. HR, hazard ratio; SGA, small for gestational age. (DOCX) [file pmed.1003255.s009.docx]

**S7 Table. Hazard ratios for maternal chronic kidney disease by history of preeclampsia and small for gestational age, among women who first live birth occurred between 1973 and 2012 in Sweden with and without those who later developed postpartum hypertension (n=1,924,409)**

|  | | **n** | **Fully adjusted** | **Excluding women with postpartum hypertension** |
| --- | --- | --- | --- | --- |
|  | |  | **HR (95% CI*)** | **HR (95% CI*)** |
| **Overall CKD** | |  |  |  |
| No preeclampsia, no SGA | | 15,783 | 1.0 | 1.0 |
| Preeclampsia only | | 1,318 | 1.96 (1.85-2.08) | 1.73 (1.62-1.85) |
| SGA only | | 1,150 | 1.32 (1.24-1.40) | 1.33 (1.25-1.42) |
| Preeclampsia & SGA | | 226 | 1.95 (1.71-2.22) | 1.59 (1.36-1.86) |
| **1.** | **Tubulointerstitial CKD** |  |  |  |
|  | No preeclampsia, no SGA | 2,458 | 1.0 | 1.0 |
|  | Preeclampsia only | 157 | 1.47 (1.25-1.73) | 1.29 (1.08-1.54) |
|  | SGA only | 171 | 1.30 (1.11-1.51) | 1.31 (1.11-1.53) |
|  | Preeclampsia & SGA | 27 | 1.41 (0.97-2.07) | 1.28 (0.85-1.94) |
| **2.** | **Glomerular/proteinuric CKD** |  |  |  |
|  | No preeclampsia, no SGA | 5,151 | 1.0 | 1.0 |
|  | Preeclampsia only | 425 | 2.11 (1.90-2.33) | 1.99 (1.79-2.22) |
|  | SGA only | 417 | 1.46 (1.32-1.62) | 1.47 (1.33-1.64) |
|  | Preeclampsia & SGA | 75 | 2.16 (1.71-2.71) | 2.09 (1.63-2.66) |
| **3.** | **Hypertensive CKD** |  |  |  |
|  | No preeclampsia, no SGA | 610 | 1.0 | *-* |
|  | Preeclampsia only | 104 | 3.60 (2.90-4.47) | *-* |
|  | SGA only | 57 | 1.54 (1.17-2.02) | *-* |
|  | Preeclampsia & SGA | 26 | 5.23 (3.51-7.79) | *-* |
| **4.** | **Diabetic CKD** |  |  |  |
|  | No preeclampsia, no SGA | 954 | 1.0 | 1.0 |
|  | Preeclampsia only | 189 | 4.03 (3.42-4.74) | 4.31 (3.54-5.24) |
|  | SGA only | 57 | 1.05 (0.80-1.37) | 1.04 (0.75-1.46) |
|  | Preeclampsia & SGA | 26 | 3.49 (2.36-5.16) | 3.29 (2.00-5.42) |
| **5.** | **Other/unspecified CKD** |  |  |  |
|  | No preeclampsia, no SGA | 6,611 | 1.0 | 1.0 |
|  | Preeclampsia only | 443 | 1.54 (1.40-1.70) | 1.38 (1.24-1.54) |
|  | SGA only | 448 | 1.24 (1.13-1.37) | 1.25 (1.13-1.38) |
|  | Preeclampsia & SGA | 72 | 1.46 (1.15-1.84) | 1.13 (0.85-1.49) |

Hazard ratios represent separate Cox regression models for associations between preeclampsia and maternal chronic kidney disease. Preeclampsia was a time-dependent variable.

Fully adjusted models controlled for maternal age, country of origin, education level, parity, maternal BMI, smoking in pregnancy, exposure to gestational diabetes, and exposure to gestational hypertension. Models were stratified by year of delivery. Abbreviations: CI, confidence interval; HR, hazard ratio; SGA, small for gestational age. *All p<0.001
