# Supplementary material for: Hypertensive disorders of pregnancy and the risk of chronic kidney disease: A Swedish registry-based cohort study
Source: PLoS Med. 2020 Aug 14;17(8):e1003255. doi: 10.1371/journal.pmed.1003255 (PMC7428061; doi:10.1371/journal.pmed.1003255)
Supplement: S8 Table — HRs represent separate Cox regression models for associations between preeclampsia and maternal CKD. Preeclampsia was a time-dependent variable. Fully adjusted models controlled for maternal age, country of origin, education level, parity, maternal BMI, smoking in pregnancy, exposure to gestational diabetes, and exposure to gestational hypertension. Models were stratified by year of delivery. *All p < 0.001. **Exact number not reported as cell count ≤ 5. HR, hazard ratio; ne, not estimable; SGA, small for gestational age. (DOCX) [file pmed.1003255.s010.docx]

**S8 Table. Hazard ratios for maternal chronic kidney disease by history of preeclampsia and preterm delivery, among women who first live birth occurred between 1973 and 2012 in Sweden with and without those who later developed postpartum hypertension (n=1,924,409)**

|  | | **N** | **Fully adjusted** | **Excluding women with postpartum hypertension** |
| --- | --- | --- | --- | --- |
|  | |  | **HR (95% CI*)** | **HR (95% CI*)** |
| **Overall CKD** | |  |  |  |
| Term delivery, no preeclampsia | | 15,134 | 1.0 | 1.0 |
| Moderate preterm delivery, no preeclampsia | | 1,552 | 1.46 (1.39-1.54) | 1.44 (1.36-1.52) |
| Very/Extremely preterm delivery, no preeclampsia | | 247 | 1.63 (1.44-1.85) | 1.64 (1.44-1.88) |
| Term delivery + Preeclampsia | | 1,196 | 1.87 (1.76-1.99) | 1.64 (1.52-1.75) |
| Moderate preterm delivery + Preeclampsia | | 276 | 2.52 (2.23-2.85) | 2.15 (1.86-2.48) |
| Very/Extremely preterm delivery + Preeclampsia | | 72 | 3.19 (2.53-4.02) | 2.66 (2.03-3.48) |
| **1.** | **Tubulointerstitial CKD** |  |  |  |
|  | Term delivery, no preeclampsia | 2,365 | 1.0 | 1.0 |
|  | Moderate preterm delivery, no preeclampsia | 220 | 1.26 (1.09-1.44) | 1.25 (1.08-1.44) |
|  | Very/Extremely preterm delivery, no preeclampsia | 44 | 1.75 (1.30-2.36) | 1.68 (1.23-2.29) |
|  | Term delivery + Preeclampsia | 146 | 1.45 (1.22-1.72) | 1.25 (1.04-1.51) |
|  | Moderate preterm delivery + Preeclampsia | 24 | 1.37 (0.91-2.05) | 1.20 (0.77-1.87) |
|  | Very/Extremely preterm delivery + Preeclampsia | 14 | 3.27 (1.93-5.54) | 3.16 (1.83-5.45) |
| **2.** | **Glomerular/proteinuric CKD** |  |  |  |
|  | Term delivery, no preeclampsia | 4,982 | 1.0 | 1.0 |
|  | Moderate preterm delivery, no preeclampsia | 508 | 1.48 (1.35-1.62) | 1.50 (1.36-1.65) |
|  | Very/Extremely preterm delivery, no preeclampsia | 78 | 1.59 (1.27-1.98) | 1.66 (1.33-2.09) |
|  | Term delivery + Preeclampsia | 393 | 1.98 (1.78-2.20) | 1.90 (1.70-2.13) |
|  | Moderate preterm delivery + Preeclampsia | 81 | 2.69 (2.16-3.35) | 2.63 (2.08-3.32) |
|  | Very/Extremely preterm delivery + Preeclampsia | 26 | 3.88 (2.64-5.71) | 2.93 (1.85-4.66) |
| **3.** | **Hypertensive CKD** |  |  |  |
|  | Term delivery, no preeclampsia | 573 | 1.0 | *-* |
|  | Moderate preterm delivery, no preeclampsia | 77 | 1.92 (1.51-2.45) | *-* |
|  | Very/Extremely preterm delivery, no preeclampsia | 17 | 2.98 (1.84-4.84) | *-* |
|  | Term delivery + Preeclampsia | 104 | 3.65 (2.92-4.55) | *-* |
|  | Moderate preterm delivery + Preeclampsia | 22 | 5.47 (3.55-8.43) | *-* |
|  | Very/Extremely preterm delivery + Preeclampsia | ** | 5.74 (2.14-15.40) | *-* |
| **4.** | **Diabetic CKD** |  |  |  |
|  | Term delivery, no preeclampsia | 839 | 1.0 | 1.0 |
|  | Moderate preterm delivery, no preeclampsia | 150 | 2.54 (2.13-3.03) | 2.79 (2.26-3.44) |
|  | Very/Extremely preterm delivery, no preeclampsia | 22 | 2.55 (1.66-3.89) | 3.19 (1.99-5.12) |
|  | Term delivery + Preeclampsia | 155 | 3.69 (3.08-4.41) | 3.88 (3.11-4.84) |
|  | Moderate preterm delivery + Preeclampsia | 56 | 8.80 (6.67-11.60) | 10.18 (7.38-14.04) |
|  | Very/Extremely preterm delivery + Preeclampsia | ** | 3.18 (1.19-8.51) | ne |
| **5.** | **Other/unspecified CKD** |  |  |  |
|  | Term delivery, no preeclampsia | 6,375 | 1.0 | 1.0 |
|  | Moderate preterm delivery, no preeclampsia | 597 | 1.34 (1.24-1.46) | 1.33 (1.21-1.45) |
|  | Very/Extremely preterm delivery, no preeclampsia | 86 | 1.37 (1.10-1.69) | 1.43 (1.15-1.79) |
|  | Term delivery + Preeclampsia | 398 | 1.50 (1.36-1.67) | 1.33 (1.18-1.49) |
|  | Moderate preterm delivery + Preeclampsia | 93 | 1.68 (1.33-2.12) | 1.37 (1.05-1.80) |
|  | Very/Extremely preterm delivery + Preeclampsia | 24 | 2.50 (1.68-3.74) | 2.16 (1.38-3.40) |

Hazard ratios represent separate Cox regression models for associations between preeclampsia and maternal chronic kidney disease. Preeclampsia was a time-dependent variable.

Fully adjusted models controlled for maternal age, country of origin, education level, parity, maternal BMI, smoking in pregnancy, exposure to gestational diabetes, and exposure to gestational hypertension. Models were stratified by year of delivery. Abbreviations: CI, confidence interval; HR, hazard ratio; SGA, small for gestational age

*All p<0.001

** Exact number not reported as cell count ≤5

ne, not estimable
