# Supplementary material for: Hypertensive disorders of pregnancy and the risk of chronic kidney disease: A Swedish registry-based cohort study
Source: PLoS Med. 2020 Aug 14;17(8):e1003255. doi: 10.1371/journal.pmed.1003255 (PMC7428061; doi:10.1371/journal.pmed.1003255)
Supplement: S9 Table — (DOCX) [file pmed.1003255.s011.docx]

**S9 Table. Hazard ratios for maternal chronic kidney disease by history of recurrent preeclampsia, among women whose first live birth occurred between 1973 and 2012 in Sweden with and without those who later developed postpartum hypertension (n=1,924,409)**

|  | | **N** | **Fully adjusted** | **Excluding women with postpartum hypertension** |
| --- | --- | --- | --- | --- |
|  | |  | **HR (95% CI)** | **HR (95% CI)** |
| **Overall CKD** | |  |  |  |
| Two pregnancies without preeclampsia | | 6,326 | 1.0 | 1.0 |
| Two pregnancies, one episode preeclampsia | | 551 | 1.82 (1.66-1.99) | 1.61 (1.45-1.78) |
| Two pregnancies, two episodes preeclampsia | | 90 | 2.64 (2.14-3.25) | 2.21 (1.73-2.82) |
| **1.** | **Tubulointerstitial CKD** |  |  |  |
|  | Two pregnancies without preeclampsia | 1,054 | 1.0 | 1.0 |
|  | Two pregnancies, one episode preeclampsia | 78 | 1.58 (1.25-1.99) | 1.47 (1.14-1.88) |
|  | Two pregnancies, two episodes preeclampsia | 10 | 1.74 (0.93-3.25) | 0.74 (0.28-1.98) |
| **2.** | **Glomerular/proteinuric CKD** |  |  |  |
|  | Two pregnancies without preeclampsia | 2,062 | 1.0 | 1.0 |
|  | Two pregnancies, one episode preeclampsia | 183 | 2.02 (1.73-2.35) | 1.91 (1.62-2.25) |
|  | Two pregnancies, two episodes preeclampsia | 35 | 3.42 (2.44-4.78) | 3.31 (2.32-4.72) |
| **3.** | **Hypertensive CKD** |  |  |  |
|  | Two pregnancies without preeclampsia | 217 | 1.0 | *-* |
|  | Two pregnancies, one episode preeclampsia | 38 | 3.23 (2.25-4.63) | *-* |
|  | Two pregnancies, two episodes preeclampsia | 7 | 5.30 (2.47-11.36) | *-* |
| **4.** | **Diabetic CKD** |  |  |  |
|  | Two pregnancies without preeclampsia | 340 | 1.0 | 1.0 |
|  | Two pregnancies, one episode preeclampsia | 73 | 3.74 (2.88-4.86) | 3.56 (2.59-4.90) |
|  | Two pregnancies, two episodes preeclampsia | 14 | 6.80 (3.96-11.68) | 7.70 (4.17-14.19) |
| **5.** | **Other/unspecified CKD** |  |  |  |
|  | Two pregnancies without preeclampsia | 2,651 | 1.0 | 1.0 |
|  | Two pregnancies, one episode preeclampsia | 179 | 1.36 (1.16-1.58) | 1.21 (1.02-1.43) |
|  | Two pregnancies, two episodes preeclampsia | 24 | 1.64 (1.10-2.46) | 1.45 (0.92-2.28) |
