# Supplementary material for: Hypertensive disorders of pregnancy and the risk of chronic kidney disease: A Swedish registry-based cohort study
Source: PLoS Med. 2020 Aug 14;17(8):e1003255. doi: 10.1371/journal.pmed.1003255 (PMC7428061; doi:10.1371/journal.pmed.1003255)
Supplement: S10 Table — HRs represent separate Cox regression models for associations between preeclampsia and maternal CKD. Gestational hypertension was a time-dependent variable. Fully adjusted models controlled for maternal age, country of origin, education level, parity, maternal BMI, smoking in pregnancy, exposure to gestational diabetes, and exposure to preeclampsia. Models were stratified by year of delivery. HR, hazard ratio; SGA, small for gestational age. (DOCX) [file pmed.1003255.s012.docx]

**S10 Table. Hazard ratios for maternal chronic kidney disease by history of gestational hypertension, among women whose first live birth occurred between 1973 and 2012 in Sweden (n=1,924,409)**

|  | | **n** | **Age-adjusted** | **Fully adjusted** |
| --- | --- | --- | --- | --- |
|  | |  | **HR (95% CI)** | **HR (95% CI)** |
| **Overall CKD** | |  |  |  |
| No gestational hypertension | | 17,734 | 1.0 | 1.0 |
| Gestational hypertension | | 743 | 1.71 (1.58-1.84) | 1.49 (1.38-1.61) |
| **1.** | **Tubulointerstitial CKD** |  |  |  |
|  | No gestational hypertension | 2,731 | 1.0 | 1.0 |
|  | Gestational hypertension | 90 | 1.43 (1.16-1.77) | 1.34 (1.08-1.65) |
| **2.** | **Glomerular/proteinuric CKD** |  |  |  |
|  | No gestational hypertension | 5,897 | 1.0 | 1.0 |
|  | Gestational hypertension | 171 | 1.20 (1.03-1.40) | 1.06 (0.91-1.24) |
| **3.** | **Hypertensive CKD** |  |  |  |
|  | No gestational hypertension | 714 | 1.0 | 1.0 |
|  | Gestational hypertension | 83 | 4.36 (3.47-5.48) | 3.13 (2.47-3.97) |
| **4.** | **Diabetic CKD** |  |  |  |
|  | No gestational hypertension | 1,142 | 1.0 | 1.0 |
|  | Gestational hypertension | 84 | 3.00 (2.40-3.75) | 1.96 (1.56-2.47) |
| **5.** | **Other/unspecified CKD** |  |  |  |
|  | No gestational hypertension | 7,250 | 1.0 | 1.0 |
|  | Gestational hypertension | 315 | 1.71 (1.53-1.92) | 1.57 (1.40-1.76) |

Hazard ratios represent separate Cox regression models for associations between preeclampsia and maternal chronic kidney disease. Gestational hypertension was a time-dependent variable.

Fully adjusted models controlled for maternal age, country of origin, education level, parity, maternal BMI, smoking in pregnancy, exposure to gestational diabetes, and exposure to preeclampsia. Models were stratified by year of delivery. Abbreviations: CI, confidence interval; HR, hazard ratio; SGA, small for gestational age
