# Supplementary material for: Hypertensive disorders of pregnancy and the risk of chronic kidney disease: A Swedish registry-based cohort study
Source: PLoS Med. 2020 Aug 14;17(8):e1003255. doi: 10.1371/journal.pmed.1003255 (PMC7428061; doi:10.1371/journal.pmed.1003255)
Supplement: Attachment — Submitted filename: PLOS_Medicine Response Letter 300520.docx [file pmed.1003255.s014.docx]

**RESPONSE TO REVIEWERS**

**A. COMMENTS FROM THE EDITORS**

**(1) *The abstract should have a minor restructure, with the headings Background, Methods and Findings, Conclusions (Methods and Findings are a single combined section).**

Thank you for this suggestion. We have now restructured the abstract accordingly.

**(2) *In the last sentence of the Abstract Methods and Findings section, would suggest including a brief description of the main limitation(s) of the study's methodology.**

We have now included the limitations relating to potential under-diagnosis of CKD in the Swedish national registers, including the possibility that some women may have been too young to have developed symptomatic CKD despite the long follow-up time. We have also included under-reporting of postpartum hypertension as a potential limitation.

**(3) *At this stage, we ask that you include a short, non-technical Author Summary of your research to make findings accessible to a wide audience that includes both scientists and non-scientists. The Author Summary should immediately follow the Abstract in your revised manuscript. This text is subject to editorial change and should be distinct from the scientific abstract. Please see our author guidelines for more information:** <https://journals.plos.org/plosmedicine/s/revising-your-manuscript#loc-author-summary>

We have now included a short Author Summary immediately after the Abstract in the revised manuscript.

**(4) *Currently, the STROBE guideline is used to support study reporting (and provided as supporting information), this is good, but would also suggest including a mention of this somewhere in the Methods section (eg stating - "This study is reported as per the Strengthening the Reporting of Observational Studies in Epidemiology (STROBE) guideline (then call out the supporting information file)".**

We have now included the suggested amendment in the Methods section (lines 161-162) and we have included the STROBE guideline in the Supplement. However, the exact pages referred to within the STROBE guideline may not correspond to the page numbers ‘in print’ if accepted for publication. We would be grateful for editorial assistance in this regard if appropriate.

**(5) *Would also suggest clarifying in the Methods section of the paper whether the study had a prospective protocol or analysis plan. Please state this (either way) early in the Methods section.**

i. **If a prospective analysis plan (from your funding proposal, IRB or other ethics committee submission, study protocol, or other planning document written before analyzing the data) was used in designing the study, please include the relevant prospectively written document with your revised manuscript as a Supporting Information file to be published alongside your study, and cite it in the Methods section. A legend for this file should be included at the end of your manuscript.**
ii. **If no such document exists, please make sure that the Methods section transparently describes when analyses were planned, and when/why any data-driven changes to analyses took place.**
iii. **In either case, changes in the analysis-- including those made in response to peer review comments-- should be identified as such in the Methods section of the paper, with rationale.**

Thank you for this suggestion. Our analyses were pre-planned and we have now made this clearer in the Methods section. We have clarified that our selection of CKD etiologies and selection of covariates was done *a priori*. Our sensitivity analyses were also pre-planned and we have made this clearer in the text. This was a necessary step for us to ensure we avoided any overlap with our previous work on preterm delivery and long-term risk of maternal CKD (recently published in BMC Medicine, April 2020). Unfortunately, we do not have a document outlining this prospective analysis plan in detail.

-----------------------------------------------------------

**B. COMMENTS FROM REVIEWER 1**

 **(6) The study was limited to women having live births. When estimating the effect of HDP on CKD, this offers 2 challenges: 1) there may be shared predictors of live birth (vs still birth or miscarriage) and CKD, which mean that the incidence of CKD is higher/lower among women who have at least one live birth than the total population of women who have ever been pregnancy (which may alter the estimated HR);** **and 2) if HPD affect the probability of having a live birth AND there is a common cause of live birth and CKD, restricting the study population to those with live births will cause a collider stratification bias (see Cole SR, Platt RW, Schisterman EF, Chu H, Westreich D, Richardson D, Poole C. Illustrating bias due to conditioning on a collider. International journal of epidemiology. 2010 Apr 1;39(2):417-20.). To avoid the first concern, the manuscript should clearly state that the relevant target population of interest is women who have had at least one live birth, rather than all women who have been pregnant. To avoid the second concern, I recommend against using causal language.**

We would like to thank the reviewer for this important observation and we are in agreement with this. We also thank the reviewer for their pragmatic suggestions for improvement. We have now endeavoured to ensure that the manuscript clearly states that the target population of interest is women who have had at least one live birth, rather than all women who have ever been pregnant. These amendments have been made in the Abstract, Results section (line 275), and Table headings.

We have also edited/improved our language to avoid drawing causal inferences. Examples of this may be seen in multiple areas in the Discussion (e.g. Lines 341-342 and 371-372) and in the Conclusion (Line 439) as well as in the Abstract.

**(7) The study excluded CKD diagnoses that occurred before a woman's last delivery. On page 7, line 178, the manuscript states that "we only considered women who were diagnosed with CKD at least 3 months after the last pregnancy." This raises several questions and concerns. First, what happened to women who had a CKD diagnosis and then had another pregnancy/delivery? Moreover, excluding these diagnoses essentially makes the time between first and last delivery "immortal", which opens the door to a variety of immortal time biases. For example, this approach may omit women experiencing such a strong effect of  HDP that they are diagnosed with CKD just 6 months after their first delivery (if they later go on to deliver 2 more children later in life). Typically, any outcome definition that requires looking into the future to classify patients as cases or not opens the door to bias. This is particularly true if there may be common causes of future pregnancies and CKD.**

Women who had a CKD diagnosis were censored on the date of their diagnosis. This was outlined in the Methods section previously but we have now made this clearer in the text (Lines 213-214 & Lines 246-248). If a woman was diagnosed with CKD more than 3 months after her first delivery but before the date of her second delivery, she was only followed up until the date of her CKD diagnosis and censored at that point. She was not excluded from the analysis entirely, but only contributed person-time until the date of her CKD diagnosis (or censoring for any other reason), thus any subsequent deliveries (after CKD diagnosis) were not included. We cannot exclude the possibility of immortal time bias in the study and this is an inherent limitation of the work. We have now acknowledged this as a limitation in the Discussion section (Lines 410-411).

**(8) Handling of competing events. Throughout, the analysis censored patients at death (I believe). If this is true, it should be stated explicitly. Moreover, a statement should be added to the definition of the parameter of interest and the interpretation of the results that the reported HRs are the HRs that would be seen could mortality be eliminated during the study period (see Austin PC, Lee DS, Fine JP. Introduction to the analysis of survival data in the presence of competing risks. Circulation. 2016 Feb 9;133(6):601-9). I also recommend presenting the results alongside results reporting overall mortality during the study period and, if overall mortality is not negligible, mortality by exposure group.**

Thank you for this suggestion. The reviewer is correct, that we censored patients at death. We mentioned this in the first paragraph of the Methods (Lines 145-147) and we have now re-stated this explicitly further in the Methods section (Line 248). We have also added a statement in the Methods section that the reported hazard ratios are those that would be observed if mortality could be eliminated during the study period (Lines 248-250).

Overall mortality during the study period was 2.8% (n=53,265) and we have now reported this in the Results section as requested. This was reasonably low, but this is perhaps unsurprising since the mean age at first delivery was 27.0 years and median follow-up time was 20.7 years, thus we had a young, relatively healthy sample. We undertook a crude analysis of mortality by exposure group, and it was marginally higher among women exposed to preeclampsia (2.86% vs. 2.76%) but the difference was not statistically significant (p=0.076).

**Specific points:
(9)      Page 4, line 113: here, or in the statistical analysis section, please make it clear that deaths were censored (or clarify if this was not the case).**

This has now been clarified in the Statistical Analysis section (Line 248)

**(10)      Page 4, line 115: here, or in the results, please report how many were excluded based on each criterion.**

This information has now been added as requested.

**(11)      Page 6, line 160: minor point, but the parenthetical here I believe should read (32 weeks to 36 weeks + 6 days) to avoid confusion**

This change has now been incorporated

**(12)      Page 6, line 165: please add a sentence of phrase here stating that this analysis was conducted only among women with exactly 2 deliveries. I was left wondering what the exposure definition was for women with >2 deliveries and did not understand that this was conducted among a subset until the Results.**

We have now improved the language to make this clearer for the readers.

**(13)      Page 7, line 189: how were covariates selected?**

We have now clarified that covariates were selected *a priori* (Line 227). This was based on multiple factors including our reading of previous literature, discussions between the research team and clinical colleagues around potential biological mechanisms, and the drafting of directed acyclic graphs to avoid over-adjusting on potential mediators.

**(14)      Page 7, line 195: the missing indicator method to account for missing data has been shown to produce biased results in many settings (see e.g., Greenland S, Finkle WD. A critical look at methods for handling missing covariates in epidemiologic regression analyses. American journal of epidemiology. 1995 Dec 15;142(12):1255-64.). Are there features of the current analysis that lead us to believe that it will work well here? If so, those should be stated.**

We agree with the reviewer that using indicators for missing values may produce biased results. Although the Swedish national registers generally contain very few missing values, data on maternal BMI and smoking status are exceptions to this as shown in Table 1. As described in our Methods (Lines 232-234) and Discussion (Lines 423-425), these variables were only collected from 1982 onwards, and were more complete from 1987 onwards. For these reasons we conducted sensitivity analyses from 1987 onwards to check for consistency with our overall results. The results of our sensitivity analyses were not substantially different and the 95% confidence intervals overlapped with the effect estimates obtained from 1973 onwards.

**(15)     Page 8, line 200: from my understanding of the exposure definitions, women were considered to have gestational hypertension if they had a subset of the criteria for preeclampsia. If this is true, wouldn't these variables be perfectly collinear?**

Gestational hypertension and preeclampsia are two distinct clinical entities, and they are mutually exclusive diagnoses in any given pregnancy (i.e. it is not possible to be diagnosed with both preeclampsia and gestational hypertension simultaneously). For the purposes of the study period 1973 to 2013, the main distinguishing feature between these diagnoses was the presence or absence of proteinuria, although preeclampsia may also have been distinguished by other features of end-organ damage (e.g. liver dysfunction, renal dysfunction). The reviewer is correct that there are similarities in their presentation, but women who had gestational hypertension were not a subset of those who had preeclampsia. Since they tend to affect different women, collinearity was not a particular concern.

**(16)     Page 8, line 210: in models 2 through 4, the model form should be explained. Specifically, how was SGA modeled in model 2? Did the models include interaction terms between preeclampsia and SGA / preterm delivery?**

We did not provide a detailed explanation for this in the Statistical Analysis section as we felt it may have led to excessive repetition with the information provided in the earlier section on Exposure variables (Lines 189-201). For example, for model 2 (SGA), a series of dummy variables were included to represent non-overlapping scenarios: (i) history of preeclampsia (ii) history of SGA (iii) history of preeclampsia and SGA (in same pregnancy). SGA was treated as a time-dependent variable. These models did not include interaction terms between preeclampsia and SGA.

Preterm delivery was defined as any delivery before 37 weeks gestation. Preterm deliveries were categorised as moderate (32 weeks to 36+6 weeks), very (28 weeks to 31+6 weeks) or extremely preterm (<28 weeks gestation). The latter two categories (very/extremely preterm) were combined in analyses due to small numbers. Maternal exposure to preterm delivery was time-dependent, and was allowed to change multiple times across different pregnancies, but exposure status was always based on the earliest gestation of any previous delivery. Again, we created non-overlapping scenarios using multiple dummy variables for Model 3 (preterm delivery) but we did not include interaction terms between preeclampsia and preterm delivery.

**(17)     Page 9, line 239: how many CKDs were excluded because they occurred before woman's last delivery?**

As mentioned above, women were not excluded from the analysis on the basis of CKD diagnosis before their last delivery. If a woman was diagnosed with CKD at least 3 months after her first delivery, she was included in the dataset. However, some women were censored between the first and last delivery if newly diagnosed with CKD during that time. Such women were included in the overall dataset but they stopped contributing person-time after the date they were first diagnosed with CKD. Thus, any subsequent live births (occurring after CKD diagnosis) were not counted towards follow-up because the mother had already developed the outcome CKD. We have now endeavoured to make this clearer from the text (Lines 246-248).

**(18)     Table 1: Is it correct to assume those in the "Preeclampsia" column were those who ever had a diagnosis of preeclampsia? If so, this should be noted (and if not, it should be clarified).**

Yes, this is correct. We have now clarified this in the footnote of the table. Thank you for this suggestion.

**(19)     Page 12, line 251: should the first sentence read "Women who had ever experienced preeclampsia…" (rather than "ever experienced") to make it clear that preeclampsia exposure was time varying?**

Yes, we have now incorporated this change.

**(20)     Page 21, line 357: should this be "incidence" rather than "prevalence"?**

Thank you for this observation. We agree that “incidence” is more suitable here and we have made this amendment.

-----------------------------------------------------------

**C. COMMENTS FROM REVIEWER 3**

**(21) They do not have long-term blood pressure value of patients and therefor can not conclude that screening for renal function or albuminuria would have any additional value after preeclampsia. Screening for hypertension after preeclampsia and correcting for this while making statement on renal follow-up is essential**

Thank you for this suggestion. We acknowledge that this is a limitation because we do not have long-term blood pressure values for these women. The additional value of screening for albuminuria is indeed uncertain, and may even depend on the etiology of CKD. We have now included this point in the Discussion (Lines 385-388).

**(22) Mechanisms of renal damage after preeclampsia can be multi-factorial. So far only marginal difference in sFlt1 are found as reviewed by Hypertension. 2016;68:00-00. DOI: 10.1161/HYPERTENSIONAHA.116.07907. NRF-2 is not a well know factor associated with glomerular damage in preeclampsia. Changes in the RAAS system, metabolic system and other factors causing endothelial dysfunction could be involved.**

Thank you for this observation. We agree that this is a complex, multifactorial issue. Although it is beyond the scope of the paper to give a very detailed overview of the potential mechanisms, we have now acknowledged that changes in the RAAS system, metabolic system and other factors causing endothelial dysfunction may also be involved (Lines 118-119).

-----------------------------------------------------------

**D. COMMENTS FROM REVIEWER 3**

**(23) This is an interesting and comprehensive analysis which has examined the long-term risk of CKD in women who developed hypertensive disorders in pregnancy. It represents a large and well curated dataset and provides important information which will inform the care and counselling of women who develop pregnancy complications. I have a number of questions listed below which I think could be clarified in the manuscript. Overall the data is well-described and clearly presented.**

Thank you for this positive feedback on our work.

**(24) Why were still births excluded? I accept that sometimes difficult to know the cause but stillbirths in the context of preeclampsia/SGA (approx. 40% of stillbirths) should be included?**

Thank you for this suggestion. We excluded women who had stillbirths because they are more likely to experience preeclampsia compared with women who only have live births. In addition, we have recently analysed national registry data from Sweden and reported that women who experience stillbirths are at higher risk of CKD compared with women who only have live births. We decided to exclude these women at baseline to avoid potential confounding. We have now included a summary of this rationale in the revised manuscript (Lines 148-151).

**(25) What was the comparator group(s) in the recurrent PE model? Are women with more than two pregnancies excluded from this analysis - needs clarification.**

We apologise if this was unclear previously. Yes, women with more than two pregnancies were excluded from this analysis due to some limitations of the dataset and to ensure that the data were still time-varying. We have now clarified in the Methods section that for this analysis only, we restricted the dataset to women who had exactly two live births (Lines 202-204 and Lines 255-257).

**(26) Definition (coding) of gestational hypertension? - women who only developed hypertension immediately after birth (ie within 6 weeks) or women who developed hypertension anytime after or between pregnancies?**

The definition of gestational hypertension was based on new-onset hypertension (without proteinuria) after 20 weeks gestation and up to the date of delivery in women with no prior diagnosis of chronic hypertension. This did not include women with de novo hypertension after the date of delivery (even if it occurred within the first 6 weeks postpartum). This has now been clarified in the text (Line 207). Women who developed hypertension outside of pregnancy, or between pregnancies, were either excluded with chronic hypertension (at baseline) or considered to have developed postpartum hypertension.

**(27) It would seem from the discussion that there is a distinct possibility that the diagnosis of post partum hypertension is under reported. This under reporting may significantly attenuate the association between pregnancy disease and CKD specifically - ie the association may be attributable to persistent hypertension (and/or diabetes) than pre-eclampsia per se? Should this perhaps be acknowledged in the abstract?**

The reviewer is correct that there is a strong possibility that postpartum hypertension may be under-reported, particularly among those who were never hospitalised during the follow-up period. We have now acknowledged this in the abstract. It is uncertain to what extent the association between preeclampsia and CKD may be mediated through persistent hypertension (and/or diabetes). We have also acknowledged the need for more focused research on this issue in the Discussion (Lines 433-436)

**(28) Whilst I understand the reason for excluding baseline hypertension diagnoses from the analysis - I wonder whether if this data is available whether it would be useful to compare the long term risk of CKD in this group of women which would provide a useful context comparison to the rates of CKD in women with de novo hypertension developing in pregnancy. I suspect this data is not available though?**

Thank you for this suggestion. We agree with the reviewer that this would be an interesting comparison. However, we have not undertaken this analysis for two reasons. Firstly, the registry datasets available to our team are somewhat limited as they only reflect hospital-diagnosed chronic hypertension. Secondly, another PhD student in our Department is already undertaking a more comprehensive analysis of women with pre-pregnancy hypertension and measuring associations with long-term health outcomes, including the long-term risk of CKD and ESKD. We have not explored this analysis further to avoid any direct overlap or duplication with her work.

**(29) Why do such a large number of CKD cases have non-specified CKD. Does the coding account for AKI related to another event which subsequently resolves?**

It is unfortunate that such a large number of CKD cases have non-specified CKD. We used broad classification categories based on recommendations from the National Kidney Foundation and *a priori* discussion with Consultant Nephrologists. The high proportion of other/unspecified CKD cases is partly due to data quality; very often in the National Patient Register patients were described as having a diagnosis of CKD with no detail provided on the underlying etiology. For these cases, we had no alternative but to describe them as unspecified CKD. We did not include patients who were diagnosed with AKI, irrespective of the cause or outcome of AKI.

**(30) Are the differences in time course significant? Should the different patterns of presentation of subsequent CKD be considered in follow up programmes?**

Thank you for this interesting observation. As mentioned in the Discussion, we had fewer cases of hypertensive CKD and diabetic CKD than initially expected, and the time to diagnosis was significantly longer in these women than for glomerular or tubulointerstitial CKD. Most women whose CKD is predominantly hypertensive or diabetic in etiology are diagnosed with this in later life. Thus, it is perhaps unsurprising that we had proportionately fewer cases since our cohort was relatively young (mean 27.0 years with median follow-up of 20.7 years). By contrast, many women with glomerulonephritis and tubulo-interstitial CKD may be diagnosed earlier in life. The additional value of screening for albuminuria is uncertain beyond following women up with frequent blood pressure checks, and we have acknowledged that the benefit of systematic renal monitoring (and optimal timing of same) may indeed depend on the underlying etiology of CKD (Lines 386-395).

**(31) Line 343 in discussion - what follow up would be required. Why do the authors consider this to be a conservative over estimate?**

We have now provided further detail on what follow up might entail (Lines 386-395). The information on numbers needed to detect one adverse event is based on the systematic review and meta-analysis by Covella et al.* (2019) which stated that 157 women who had preeclampsia would need to be followed up to detect one case of CKD. Their calculations were based on a pooled crude CKD prevalence of 0.64% among control participants from previous observational studies. This prevalence is likely to be considerably lower than the true prevalence of CKD, which is estimated at approx. 3% among women of childbearing age (as per Covella et al.*) and approx. 7-12% among women in the general population during the lifetime (per Hill et al., Gasparini et al., and GBD Collaboration*). For these reasons, 157 is likely to be a conservative over-estimate.

**Please see References section of manuscript for further details*

**(32) Line 344 - 7-12% in their lifetime population risk presumably includes a large amount of CKD in older age >30years away from pregnancies and not covered by the time frame of the current analysis. The lifetime risk discussed here should be the population risk within the relevant time periods covered by the analysis presented which is not 7-12%?**

Thank you for raising this point. It is true that a large number of women who develop CKD do so >30 years after their pregnancies and this does not equate to the timeframe in our analysis. We mentioned in the text that just 0.9% of women developed CKD during follow-up (Line 283), but that this is likely to be affected by under-diagnoses and under-ascertainment in the Swedish registers (Lines 408-409). Nonetheless, we think that it is relevant to consider the broader public health implications of these findings. We have now given more explicit consideration to the need to review the optimal timing of any systematic follow-up programmes - whether they comprise of blood pressure checks alone or screening for albuminuria or other markers of renal damage (Lines 393-394).

**(33) Consider changing "non-pre-eclamptic" to women who had never had pre-eclampsia? Same for "pre-eclamptic" as a term in general.**

Thank you for this suggestion. We have now changed “preeclamptic women” to “women who had preeclampsia” where possible, and only use the term “preeclamptic” to describe a pregnancy or delivery to help the flow of language in some places. We have also changed “non-preeclamptic women” to “women who never had preeclampsia”.
