# Supplementary material for: Hypertensive disorders of pregnancy and the risk of chronic kidney disease: A Swedish registry-based cohort study
Source: PLoS Med. 2020 Aug 14;17(8):e1003255. doi: 10.1371/journal.pmed.1003255 (PMC7428061; doi:10.1371/journal.pmed.1003255)
Supplement: Attachment — Submitted filename: Responses to Reviewer Comments 260620.docx [file pmed.1003255.s015.docx]

**RESPONSES TO REVIEWER COMMENTS**

Many thanks for the opportunity to make further edits and improvements to our manuscript. We are very pleased that our manuscript is deemed eligible for publication in PLOS Medicine. Please find our responses to editorial and reviewer comments below.

**A. REQUESTS FROM EDITORS**

**1. Title – please add the country setting**

We have now amended the title accordingly.

**2. Abstract and throughout, please provide p values with quantifiable data and where 95% CIs are given**

We have now provided p values throughout the manuscript.

**3. Please be more explicit in the abstract about the limitations of your study starting with ‘Limitations of the study are….’**

We have now amended the abstract accordingly.

**4. Please use square brackets for refs in the main text.**

Square brackets are now used for all references in the main text.

**5. Please break the supp files into separate ones and the STROBE needs to be provided with sections and paragraphs instead of page numbers**

The supplementary files have now been separated and the STROBE statement refers to sections and paragraphs as requested.

**6. At line 74, "In this study, we found that ... were associated ..." or similar.**

This amendment has now been incorporated.

**7. At line 102, "Our findings suggest that ..." or similar.**

This amendment has now been incorporated.

**8. Methods, was there a protocol or prespecified analysis plan?**

Although our analyses were pre-planned, unfortunately we do not have a detailed protocol or documented analysis plan which we can include as a Supplementary file. As outlined on page 2 of our previous response letter:

“Our analyses were pre-planned and we have now made this clearer in the Methods section. We have clarified that our selection of CKD etiologies and selection of covariates was done *a priori*. Our sensitivity analyses were also pre-planned and we have made this clearer in the text. This was a necessary step for us to ensure we avoided any overlap with our previous work on preterm delivery and long-term risk of maternal CKD (recently published in BMC Medicine, April 2020). Unfortunately, we do not have a document outlining this prospective analysis plan in detail.”

**B. COMMENTS FROM REVIEWERS**

 **1.      Page 11, line 286: Presumably this is time to CKD diagnosis *after first live birth*?**

Yes, this is correct. We have now amended the wording here to reflect this.

**2.      Page 6, line 174: I found this sentence confusing. I thought the current study included all women who had live singleton births during/after 1973, not limited to those with preeclampsia after 1973. I recommend rewording this sentence to clarify that analyses were repeated after restricting the entire study population to those with a first live birth from 1987 onwards.**

Thank you for this suggestion. We have now reworded the sentence accordingly.

**3.      Page 9, line 234: I recommend clarifying that "missing indicators were used to account for missing data on smoking and BMI" (as opposed to ending the sentence with "this"). As a side note, I accept the authors' logic that, because results were similar after sensitivity analysis that limited the study population to 1987 and after, when there was less missing data, the missing indicator method was unlikely to have induced substantial bias. However, I still have concerns about use of the missing indicator method in this setting and recommend adding language to the discussion acknowledging the potential for (or at least possibility of) residual bias when using this approach.**

Thank you for this suggestion. We have incorporated the amended wording in line 234 and have added a sentence to the Discussion (line 426) outlining that residual bias is still a possibility as a result of using the missing indicator method.


**4. The manuscript has been significantly improved, I have no further suggestions/comments.**

Thank you for this feedback. We agree that the manuscript has been improved considerably as a result of editorial changes and reviewers’ edits.
